# Hormetic effects of curcumin on oxidative stress injury induced by trivalent arsenic in isolated rat hepatocytes

**DOI:** 10.22038/AJP.2023.22634

**Published:** 2023

**Authors:** Marzieh Amirmostofian, Fahimeh Akbari, Mahmoud Hashemzaei, Fahimeh Safaeinejad, Kaveh Tabrizian, Halimeh Arbab, Ramin Rezaee, Shaghayegh Hemat Jouy, Vahideh Ghorani, Jafar Shahraki

**Affiliations:** 1 *Department of Pharmacology and Toxicology, Faculty of Pharmacy, Zabol University of Medical Sciences, Zabol, Iran*; 2 *UniSA Clinical and Health Sciences, Cancer Research Institute, University of South Australia, Adelaide, South Australia, Australia*; 3 *Toxicology and Addiction Research Center, Zabol University of Medical Sciences, Zabol, Iran*; 4 *Traditional Medicine and Materia Medica Research Center, Shahid Beheshti University of Medical Sciences, Tehran, Iran*; 5 *Department of English Language, Faculty of Humanities, University of Zabol, Sistan and Balouchestan, Zabol, Iran*; 6 *International UNESCO Center for Health-Related Basic Sciences and Human Nutrition, Faculty of Medicine, Mashhad University of Medical Sciences, Mashhad, Iran*; 7 *Applied Biomedical Research Center, Mashhad University of Medical Sciences, Mashhad, Iran*; 8 *Department of Exercise Physiology, Faculty of Physical Education and Sport Sciences, Central Tehran Branch, Islamic Azad University, Tehran, Iran*; 9 *Clinical Research Development Unit, Imam Reza Hospital, Faculty of Medicine, Mashhad University of Medical Sciences, Mashhad, Iran*

**Keywords:** Hepatocytes, Reactive oxygen species, Lipid peroxidation, Mitochondria

## Abstract

**Objective:**

Arsenic (As) poisoning is a worldwide public health problem. Arsenic can cause cancer, diabetes, hepatic problems, etc. Hence, we investigated possible hepatoprotective properties of curcumin against As^3+^-induced liver damages in freshly isolated rat hepatocytes.

**Materials and Methods:**

Isolation of hepatocytes was done by the two-step liver perfusion method using collagenase. The EC_50_ concentration of As^3+^ was used in toxicity assessments and curcumin (2, 5, and 10 µM) was added 15 min before As^3+^ addition to isolated hepatocytes. Curcumin impact was assessed in terms of cytotoxicity, lipid peroxidation induction, reactive oxygen species (ROS) levels, and mitochondrial membrane potential.

**Results:**

As^3+^ significantly increased cytotoxicity, malondialdehyde and ROS levels and induced mitochondrial membrane damage and hepatocyte membrane lysis after 3 hr incubation. Curcumin 2 µM significantly prevented lipid peroxidation induction, ROS formation, and mitochondrial membrane damage; while curcumin 5 µM had no apparent effect on these parameters, curcumin 10 µM potentiated them.

**Conclusion:**

Curcumin only at low doses could ameliorate oxidative stress injury induced by As^3+^ in isolated rat hepatocytes.

## Introduction

Arsenic is widely found in organic or inorganic compounds. Inorganic compounds and in particular inorganic trivalent arsenic are much more toxic than organic compounds. Arsenic contamination of air, water and soil occurs during smelting and production of energy from fossil fuels as well as production/application of arsenical pesticides (Järup, 2003 ▶; Jomova et al., 2011 ▶).

Most of general public exposures to arsenic occur via food and drinking water. Besides, in some areas, exposure to inorganic arsenic mostly occurs via drinking water (Jomova et al., 2011 ▶).

Soluble arsenic compounds can be readily absorbed through the gastrointestinal tract. Symptoms associated with the intake of inorganic arsenic include gastrointestinal complications, cardiovascular and central nervous system disturbances, bone marrow suppression, haemolysis, melanosis, hyperkeratosis, hepatotoxicity and nephrotoxicity (Balarastaghi et al., 2022 ▶; Järup, 2003 ▶). There is also an excess risk of mortality due to skin, lung, bladder, kidney, liver and prostate cancers amongst populations that are orally exposed to arsenic (Jomova et al., 2011 ▶).

The target of arsenic toxicity is the liver, and hepatomegaly, liver fibrosis and cirrhosis were reported to happen as a result of chronic arsenic exposure (Liu and Waalkes, 2008 ▶). Reactive oxygen species (ROS) insult is seen in arsenic hepatotoxicity and carcinogenesis. Following arsenic exposure, cell membrane damage, lipid peroxidation and mitochondrial membrane damage are caused by free radicals derived from superoxide radical (Jomova et al., 2011 ▶). 

Arsenic affects cell cycle, expression of growth factors, resistance towards apoptosis and DNA repair, alterations in DNA methylation, reduced surveillance of the immune system, and increased oxidative stress. Arsenic forms oxygen-based radicals (OH• and O^2^•−) under physiological conditions by directly binding critical thiols. The carcinogenicity of arsenic may be attributed to signaling pathways involving activator protein-1, nuclear factor κB, and p53 (Famurewa et al., 2022 ▶; Flora, 2011 ▶).

Natural products, mainly those obtained from dietary sources, contain diverse anti-oxidants and phytoconstituents with anti-oxidants effects and they are capable of terminating free radical chain reactions (Gülçin, 2012 ▶). They could be used as chemopreventive agents in various cancer models (Narayanankutty et al., 2021 ▶). There are many studies on the effects of plant products to treat various diseases such as arsenic-mediated toxicity (Najafi et al., 2022 ▶; Susan et al., 2019 ▶).

 Curcumin, the main polyphenol isolated from *Curcuma longa *rhizome, exhibits anti-tumoral, anti-oxidant and anti-inflammatory properties and acts as a free-radical scavenger (Hashemzaei et al., 2020 ▶; Hassani et al., 2015 ▶; Mirzaei et al., 2016 ▶; Rezaee et al., 2017 ▶; Tabrizian et al., 2019 ▶; Wilken et al., 2011 ▶). Curcumin exerts protective effects against liver diseases associated with oxidative damage suppressing the lipid peroxidation products, pro-inflammatory cytokines, and hepatic stellate cells and PI3K/Akt activation, as well as enhancing the expression of SOD (superoxide dismutase), CAT (catalase), GSH (reduced glutathione), GPx (glutathione peroxidase), GR (glutathione reductase) and Nrf2 (Farzaei et al., 2018 ▶).

The current study investigated the possible hepatoprotective properties of curcumin against damages caused by arsenic in isolated fresh rat hepatocytes.

## Materials and Methods

**Chemicals**

Arsenic trichloride (AsCl_3_), thiobarbituric acid (TBA), collagenase type IV, bovine serum albumin (BSA), trichloroacetic acid, 2ʹ,7ʹ-dichlorofluorescin diacetate (DCFH-DA), N-(2-hydroxyethyl) piperazine-N′-(2-ethanesulfonic acid) (HEPES), and trypan blue were purchased from Sigma-Aldrich (Taufkrichen, Germany). Curcumin was purchased from Mashhad University of Medical Sciences, Mashhad, Iran. 

**Hepatocytes isolation and incubation**

Male Wistar rats (weighing 280–300 g) were obtained from animal house, Zabol University of Medical Sciences, Zabol, Iran. Standard chow diet and water *ad libitum* were provided throughout the experimental period. Zabol University of Medical Sciences Ethics Committee of Animal Experimentation, approved the study. Hepatocytes were isolated using collagenase liver perfusion as described previously (Moldeus et al., 1978 ▶) and using trypan blue, cell viability was determined; cell isolation efficiency was always >90% (Hashemzaei et al., 2015 ▶). After isolation, hepatocytes at 10^6 ^cells/ml (10 ml) were suspended in Krebs–Henseleit buffer (pH 7.4) including 12.5 mM HEPES, in flasks with 95% O_2_ and 5% CO_2_ at 37^◦^C (Shahraki et al., 2013a ▶). In this study, the effects of 3-hr incubation with curcumin (2, 5, and 10 µM) on arsenic toxicity were studied in isolated rat hepatocytes. Also, As^3+ ^at EC_50_ (50 µM) was used in order to avoid toxic and non-toxic effects. Stock solutions of As^3+ ^(×100 concentrated in Krebs–Henseleit buffer) and curcumin (×250 concentrated in Krebs–Henseleit buffer) were freshly prepared prior to use (Shahraki et al., 2014 ▶). A low concentration of dimethyl sulfoxide (DMSO) was used to help curcumin dissolve in Krebs–Henseleit buffer; the level of DMSO used does not affect the studied factors. Curcumin was added 15 min before As^3+^ addition into hepatocytes and to incubate As^3+^ at the needed level, 100 µl sample of concentrated stock solution (×100 concentrated) was added to the hepatocyte suspension (10 ml). Furthermore, curcumin was incubated at required concentration by adding 40 µl sample of concentrated stock solution (×250 concentrated) to 10 ml of hepatocyte suspension. 

**Hepatocyte viability**

Trypan blue (0.2%, w/v) was used to evaluate cell viability. Here, hepatocytes suspension was blended with trypan blue as a cell stain and then, the percentage of viable cells (cells with clear cytoplasm) versus nonviable cells (cells with blue cytoplasm) was determined. Aliquots of the hepatocytes incubate were taken after 3 hr incubation (Shahraki et al., 2013a ▶). After this period of time, at least 80% of the control cells were still viable.

**Reactive oxygen species (ROS) formation**

As^3+^-induced ROS generation was measured by adding dichlorofluorescin diacetate (DCFD) to the hepatocyte incubates. After penetrating into the cells, DCFD is deacetylated to generate non-fluorescent dichlorofluorescin (DCF). Next, reaction of DCF with ROS leads to production of highly fluorescent dichlorofluorescein. To assess ROS formation, 1 ml hepatocyte samples was taken at several time points from As^3+^-treated hepatocytes and control hepatocytes and centrifuged for 1 min at 50 g. Afterwards, the cells were re-suspended using incubation buffer containing 1.6 µM DCFD and incubated at 37^◦^C for 10 min. Finally, fluorescence intensity was examined by Shimadzu Rf-5301PC fluorescence spectrophotometer (490 nm excitation and 520 nm emission wavelength) (Shahraki et al., 2014 ▶).

**Lipid peroxidation**

Lipid peroxidation induction in As^3+^-treated hepatocytes was assayed in terms of the level of thiobarbituric acid-reactive substances (TBARS) as explained previously, using a spectrophotometer at 532 nm (Hashemzaei et al., 2015 ▶).

**Mitochondrial membrane potential**

In this part, the cell suspension after centrifugation (50 g for 1 min) was re-suspended in incubation medium which contained rhodamine 123 (1.5 µM) and then, incubated for 10 min at 37°C. After repeated centrifugation, rhodamine 123 level in the incubation medium was measured using Shimadzu Rf-5301PC fluorescence spectrophotometer (at 490 nm excitation and 520 nm emission wavelength) (Shahraki et al., 2013b ▶).

**Statistical analysis**

Data is presented as mean±SD (of three replicates). We made statistical comparisons by one-way analysis of variance (ANOVA) and *post‐hoc* Tukey's test. Statistical significance was set at p<0.05.

## Results

To determine 2-hr EC_50_ (EC_50, 2hr_) of As^3+^, dose-response curves were charted for different concentrations of As^3+^. The EC_50, 2hr _concentration for As^3+^ was 50 μM. As^3+^ led to a significant increase in cytotoxicity (Table 1). However, As^3+^-induced hepatocyte membrane lysis was significantly prevented by curcumin 2 µM (p<0.05). Curcumin 5 µM produced no significant effect on As^3+^-induced hepatocyte membrane lysis. Nevertheless, curcumin 10 µM potentiated As^3+^-induced hepatocyte membrane lysis.

Compared to the control hepatocytes, As^3+^ produced a distinct increase in ROS formation but As^3+^-induced ROS formation was significantly prevented by curcumin 2 µM (p<0.05). Curcumin 5 µM had no significant effect on As^3+^-induced ROS formation, but curcumin 10 µM potentiated As^3+^-induced ROS formation (Table 2). 

**Table 1:** Effects of curcumin on As^3+^-induced cytotoxicity in rat hepatocytes

**Cytotoxicity (%) 3 h** **r**	**Addition**
18±3	Control rat hepatocytes
75±7^a^	+As^3+^ (50 µM)
89±5^b^	+ Curcumin (10 µM)
64±5	+ Curcumin (5 µM)
54±3^b^	+ Curcumin (2 µM)

Hepatocytes (10^6 ^cells/ml) were incubated in Krebs–Henseleit buffer (pH 7.4) at 37^◦^C for 3 hr following the addition of As^3+^ at EC_50, 2hr_. Cytotoxicity was determined as the percentage of cells that take up trypan blue. Values are expressed as mean±SD of three separate experiments (n=3).^a^ Significant difference in comparison with the control hepatocytes (p<0.05).^b^ Significant difference in comparison with the As^3+^-treated hepatocytes (p<0.05).

As noted in Table 3, As^3+^ produced a noticeable increase in malondialdehyde formation and lipid peroxidation level compared to the control hepatocytes. However, As^3+^-induced malondialdehyde formation was markedly prevented by curcumin 2 µM, was not affected by curcumin 5 µM but it was increased by curcumin 10 µM.

As^3+^-caused mitochondrial membrane damage was significantly inhibited by curcumin 2 µM (p<0.05, Table 4). While curcumin 5 µM showed no significant effect on As^3+^-induced hepatocyte mitochondrial membrane damage, curcumin 10 µM worsened it. Of note, curcumin 2, 5 and 10 µM did not trigger hepatocyte membrane lysis, ROS formation, lipid peroxidation or mitochondrial membrane damage in intact rat hepatocytes during the incubation time (data not shown).

**Table 2:** Effects of curcumin on As^3+^-induced ROS formation in rat hepatocytes

**Incubation time with curcumin**			**Addition**	
**60 min**	**30 min**	**15 min**		
97±8	65±7	56±9		Control rat hepatocytes
279±13^a^	266±8^a^	255±11^a^		+As^3+^ (50 µM)
217±21^b^	295±13^b ^	277±19		+ Curcumin (10 µM)
254±19	248±16	239±14		+ Curcumin (5 µM)
168±12^b^	142±7^b^	116±8^b^		+ Curcumin (2 µM)

Hepatocytes (10^6 ^cells/ml) were incubated in Krebs–Henseleit buffer (pH 7.4) at 37^◦^C for 1 hr following the addition of As^3+^ at EC_50, 2hr_. DCF formation was expressed as fluorescent intensity units. Values are expressed as mean±SD of three separate experiments (n=3).^a^ Significant difference in comparison with the control hepatocytes (p<0.05).^b^ Significant difference in comparison with the As^3+^-treated hepatocytes (p<0.05).

**Table 3:** Effects of curcumin on As^3+^-induced lipid peroxidation in rat hepatocytes

**Incubation time**		** Addition**		
**60 min**	**30 min**	**15 min**	
3.47±0.11	2.62±0.13	1.66±0.08	Control rat hepatocytes
4.74±0.12^a^	3.74±0.08^a^	3.05±0.05^a^	+As^3+^ (50 µM)
4.98±0.10^b^	3.97±0.13^b^	3.14±0.09	+ Curcumin (10 µM)
4.57±0.13	3.81±0.10	3.08±0.08	+ Curcumin (5 µM)
3.69±0.12^b^	3.08±0.14^b^	2.66±0.06^b^	+ Curcumin (2 µM)

Hepatocytes (10^6 ^cells/ml) were incubated in Krebs–Henseleit buffer (pH 7.4) at 37^◦^C for 1 hr following the addition of As^3+^ at EC_50, 2hr_. Lipid peroxidation level was determined as the difference in malondialdehyde formation between the control and As^3+^-treated cells (7). Values are expressed as mean±SD of three separate experiments (n=3).^a^ Significant difference in comparison with the control hepatocytes (p<0.05).^b^ Significant difference in comparison with the As^3+^ treated hepatocytes (p<0.05).

**Table 4:** Effects of curcumin on As^3+^-induced mitochondrial membrane potential loss in rat hepatocytes

**Incubation time**			**Addition**
9±2	5±3	2±1	Control rat hepatocytes
42±3^a^	21±4^a^	11±3^a^	+As^3+^ (50 µM)
54±5^b^	29±3^b^	12±3	+ Curcumin (10 µM)
36±5	19±4	6±3	+ Curcumin (5 µM)
18±5^b^	7±3^b^	3±2^b^	+ Curcumin (2 µM)

Hepatocytes (10^6^ cells/ml) were incubated in Krebs–Henseleit buffer pH 7.4 at 37^◦^C for 1.0 hr following the addition of EC_50, 2hr _of As^3+^. Mitochondrial membrane potential was determined as the difference in mitochondrial uptake of the rhodamine 123 between the control and treated hepatocytes and expressed as fluorescence intensity unit (10). Values are expressed as mean±SD of three separate experiments (n=3).^a^ Significant difference in comparison with the control hepatocytes (p<0.05).^b^ Significant difference in comparison with the As^3+^ treated hepatocytes (p<0.05).

## Discussion

The liver is one of the main organs where heavy metals such as arsenic accumulate and produce toxicity (García-Niño and Pedraza-Chaverrí, 2014 ▶). Oxidative stress caused by arsenic leads to DNA damage, chromosomal aberrations, neuronal and liver toxicity and cancer (Laparra et al., 2006 ▶; Stamatelos et al., 2013 ▶; Xu et al., 2008 ▶). It is clear that oxidative stress caused by arsenic weakens the antioxidant defense and induces lipid peroxidation and DNA damage (Shi et al., 2004 ▶). Suppression of the antioxidant enzymes could be mediated via several mechanisms such as mitigated activities of superoxide dismutase (SOD), catalase (CAT) and glutathione peroxidase (GPx) (De Vizcaya-Ruiz et al., 2009 ▶). Glutathione (GSH) is a major intracellular antioxidant controlled by nuclear factor-2 related erythroid factor-2 (Nrf2) (Malayil et al., 2022 ▶; Narayanankutty et al., 2019 ▶). GSH appears in the reduced form (GSH) and oxidized form (GSSG). GPx and glutathione-s-transferase (GST) conduct detoxification reactions by employing GSH and changing it into GSSG. Glutathione reductase (GR) runs the salvage pathway by changing GSSG to GSH by using NADPH and maintains the cellular GSH pool. Therefore, GSH and GSH-dependent enzymes are required to keep the normal redox balance and cell survival in stress conditions. GSH safeguards the cells against diverse free radicals including ROS, xenobiotic toxicants, heavy metals, etc. (Narayanankutty et al., 2019 ▶; Yeang et al., 2012 ▶). Glutathione (GSH) is an important factor against the harmful effects of arsenic in the cells (Hou et al., 2014 ▶). Methylated arsenic metabolites such as monomethylated arsenic are also strong inhibitors of glutathione reductase and thioredoxin reductase; this probably occurs due to interactions between arsenic and thiol groups of these enzymes. Also, arsenic reduces Nrf2 activity (Janasik et al., 2018 ▶). Cellular thiols such as glutathione to fight against ROS has been considered a therapeutic strategy against arsenic. It has been shown that N-acetylcysteine, α-lipoic acid, vitamin E, quercetin have beneficial properties against various arsenic damages *in vitro* and *in vivo*, probably by enhancing cellular GSH content (Flora, 2011 ▶).

Arsenic can alter the activity of enzymes involved in mitochondrial respiration chain, the mitochondrial permeability transition (MPT) pore opening and release of cytochrome c from mitochondria, induce loss of mitochondrial membrane potential, electron transport chain defects, and ROS production, and promote mitochondrial morphologic changes (Hou et al., 2014 ▶) all of which leading to cell death. Our data showed the cytotoxicity of arsenic in freshly isolated rat hepatocytes, compared to the control group (Table 1). 

Superoxide radical is the first ROS which is produced by arsenic (Herbert and Snow, 2012 ▶). Arsenic produces superoxide anion by induction of NADPH oxidase and increasing NADPH. Generating superoxide anion leads to formation of a cascade of secondary ROS such as H_2_O_2_ and hydroxyl radicals. Reaction of ROS with cellular targets can result in lipid peroxidation, mitochondrial and DNA damage and finally, cell death (De Vizcaya-Ruiz et al., 2009 ▶). Our study showed that arsenic significantly enhanced production of ROS, lipid peroxides levels and mitochondrial membrane damage in As^3+^-treated rat hepatocytes compared to the control hepatocytes (Tables 2, 3, and 4).

Recently, many researches assessed the potential health benefits of flavonoids on a variety of diseases that are developed by oxidative stress. Results showed that consumption of food containing antioxidants such as polyphenols and flavonoids, can be effective on reduction of oxidative damage caused by metallic elements and anthropogenic factors (Cilla et al., 2008 ▶). The protective effects of flavonoids in biological systems are ascribed to their capacity in scavenging ROS, chelating catalytic metals, boosting cellular antioxidants and other detoxification components such as glutathione, activation of antioxidant enzymes and repression of oxidants (Morisco et al., 2008 ▶).

Anti-inflammatory and antioxidant features of curcumin could potentially contribute to prevention of hepatotoxicity caused by environmental toxins. Curcumin through increment of the activity of antioxidant enzymes such as GST, SOD and CAT, cellular GSH content and prevention of lipid peroxidation, can protect the body against heavy metals’ toxicity. The protective benefits of curcumin are ascribed to its capability to clean free radicals, chelate heavy metals and activate the detoxifying enzymes through Keap1/Nrf2/ARE pathway regulation (Job et al., 2022a ▶; Job et al., 2022b ▶). In the present study, curcumin’s protective effects may be mediated via Nrf2-mediated glutathione biosynthesis, and subsequently, improvement of the intracellular GSH pool. Also, in *in vivo* and *in vitro* studies, curcumin could protect against genotoxicity, angiogenesis, skin disorder, neurotoxicity, nephrotoxicity and hepatotoxicity (García-Niño and Pedraza-Chaverrí, 2014 ▶). 

In contrast to numerous studies which showed the beneficial therapeutic properties of curcumin against a variety of pathological conditions, some studies suggested the potential toxicity of this bioactive compound. For instance, in human subjects taking curcumin at high doses for 6 months, hepatotoxicity and elevated levels of liver enzymes were observed (Baum et al., 2007 ▶). Prior studies found that at concentrations as high as 50 µM, curcumin promotes ROS generation while at low concentrations (e.g. 10 µM), curcumin usually diminishes ROS generation (Kang et al., 2005 ▶). Curcumin was shown to induce apoptosis in cancerous cells at high levels which can cause liver toxicity (Lee et al., 2013 ▶) and may cause chromosome aberrations in several mammalian cell lines. Although experimental studies showed that curcumin has antioxidant effects, higher concentrations of curcumin increased levels of intracellular ROS (Burgos‐Morón et al., 2010 ▶). In human hepatoma G2 cells, evaluation of mitochondrial and DNA damage induced by curcumin revealed that high levels of curcumin induce ROS production, lipid peroxidation and mitochondrial injury (Cao et al., 2006 ▶).

It was indicated that low concentrations of curcumin inhibit the generation of inducible nitric oxide synthase (iNOS) and at specific concentrations, curcumin protect cells from damage caused by radioactive radiation (Bengmark, 2006 ▶; Sharma et al., 2005 ▶). The results of these studies showed hepatoprotective effects of low doses of curcumin against liver damage induced by chronic alcohol intake and a high-fat diet by affecting the alcohol metabolizing enzyme activity, antioxidant properties and lipid metabolism (Lee et al., 2013 ▶).

In the current study, curcumin 2 µM protected rat hepatocytes in terms of cell death, ROS formation, lipid peroxidation, and mitochondrial membrane damage, against trivalent arsenic toxicity. While 5 µM curcumin was unable to protect rat hepatocytes from As^3+ ^toxicity, curcumin 10 µM increased cell death after 3 hr of incubation and deteriorated lipid peroxidation and mitochondrial membrane damage caused by As^3+^ after 1 hr of incubation (Tables 3 and 4). Curcumin 10 µM enhanced As^3+^-induced ROS formation after 15- and 30- min incubation; however, after 60 min of incubation, decrement of ROS levels probably due to increased number of dead cells, was observed (Table 2). Consistently, hormetic properties of curcumin were shown in diverse biomedical models (to review, see (Moghaddam et al., 2019 ▶)). 

Of note, it was shown that curcumin bioavailability following oral consumption and intraperitoneal injection is low. Hence, we examined curcumin protection on arsenic-induced hepatotoxicity using normal hepatocyte cells that were freshly isolated from rats. We observed that low-dose (2 µM) curcumin could confer protection against arsenic toxicity, but at higher doses that had shown beneficial effects in oral consumption, curcumin not only did not reduce arsenic toxicity, but also increased arsenic toxicity (10 µM). Therefore, curcumin could be toxic in doses slightly higher than the therapeutic values. It should be noted that while some studies reported toxic effects of high doses of curcumin, other studies expressed that due to low oral bioavailability, curcumin even at high doses produce no toxic effects in animals or humans (Hatcher et al., 2008 ▶). 

According to the results of our study, low-dose curcumin can alleviate As^3+ ^toxicity in the hepatocytes while at high doses, it deteriorated arsenic hepatotoxicity. Assessment of the protective or toxic effects of intravenous curcumin versus arsenic should be examined in future studies.

## Conflicts of interest

The authors have declared that there is no conflict of interest.

## References

[B1] Balarastaghi S, Rezaee R, Hayes AW, Yarmohammadi F, Karimi G (2022). Mechanisms of arsenic exposure-induced hypertension and atherosclerosis: An updated overview. Biological Trace Element Research.

[B2] Baum L, Cheung SK, Mok VC, Lam LC, Leung VP, Hui E, Ng CC, Chow M, Ho PC, Lam S (2007). Curcumin effects on blood lipid profile in a 6-month human study. Pharmacol Res.

[B3] Bengmark S (2006). Curcumin, an atoxic antioxidant and natural NFκB, cyclooxygenase-2, lipooxygenase, and inducible nitric oxide synthase inhibitor: a shield against acute and chronic diseases. J Parenter Enteral Nutr.

[B4] Burgos‐Morón E, Calderón‐Montaño JM, Salvador J, Robles A, López‐Lázaro M (2010). The dark side of curcumin. Int J Cancer.

[B5] Cao J, Jia L, Zhou H-M, Liu Y, Zhong L-F (2006). Mitochondrial and nuclear DNA damage induced by curcumin in human hepatoma G2 cells. Toxicol Sci.

[B6] Cilla A, Laparra JM, Alegria A, Barbera R, Farre R (2008). Antioxidant effect derived from bioaccessible fractions of fruit beverages against H 2 O 2-induced oxidative stress in Caco-2 cells. Food Chem.

[B7] De Vizcaya-Ruiz A, Barbier O, Ruiz-Ramos R, Cebrian ME (2009). Biomarkers of oxidative stress and damage in human populations exposed to arsenic. Mutat Res.

[B8] Famurewa AC, Maduagwuna EK, Aloke C, Azubuike-Osu SO, Narayanankutty A (2022). Virgin coconut oil ameliorates arsenic hepatorenal toxicity and NO-mediated inflammation through suppression of oxidative stress in rats. TJPS.

[B9] Farzaei MH, Zobeiri M, Parvizi F, El-Senduny FF, Marmouzi I, Coy-Barrera E, Naseri R, Nabavi SM, Rahimi R, Abdollahi M (2018). Curcumin in liver diseases: a systematic review of the cellular mechanisms of oxidative stress and clinical perspective. Nutrients.

[B10] Flora SJ (2011). Arsenic-induced oxidative stress and its reversibility. Free Radic Biol Med.

[B11] García-Niño WR, Pedraza-Chaverrí J (2014). Protective effect of curcumin against heavy metals-induced liver damage. Food Chem Toxicol.

[B12] Gülçin İ (2012). Antioxidant activity of food constituents: an overview. Arch Toxicol.

[B13] Hashemzaei M, Pourahmad J, Safaeinejad F, Tabrizian K, Akbari F, Bagheri G, Hosseini M-J, Shahraki J (2015). Antimony induces oxidative stress and cell death in normal hepatocytes. Toxicol Environ Chem.

[B14] Hashemzaei M, Tabrizian K, Alizadeh Z, Pasandideh S, Rezaee R, Mamoulakis C, Tsatsakis A, Skaperda Z, Kouretas D, Shahraki J (2020). Resveratrol, curcumin and gallic acid attenuate glyoxal-induced damage to rat renal cells. Toxicol Rep.

[B15] Hassani S, Sepand M, Jafari A, Jaafari J, Rezaee R, Zeinali M, Tavakoli F, Razavi-Azarkhiavi K (2015). Protective effects of curcumin and vitamin E against chlorpyrifos-induced lung oxidative damage. Hum Exp Toxicol.

[B16] Hatcher H, Planalp R, Cho J, Torti F, Torti S (2008). Curcumin: from ancient medicine to current clinical trials. Cell Mol Life Sci.

[B17] Herbert KJ, Snow ET (2012). Modulation of arsenic-induced epidermal growth factor receptor pathway signalling by resveratrol. Chem Biol Interact.

[B18] Hou Y, Wang Y, Wang H, Xu Y (2014). Induction of glutathione synthesis in human hepatocytes by acute and chronic arsenic exposure: Differential roles of mitogen-activated protein kinases. Toxicology.

[B19] Janasik B, Reszka E, Stanislawska M, Jablonska E, Kuras R, Wieczorek E, Malachowska B, Fendler W, Wasowicz W (2018). Effect of arsenic exposure on NRF2-KEAP1 pathway and epigenetic modification. Biol Trace Elem Res.

[B20] Järup L (2003). Hazards of heavy metal contamination. Br Med Bull.

[B21] Job JT, Rajagopal R, Alfarhan A, Kim YO, Kim H-J, Narayanankutty A (2022a). Protective effect of borassus flabellifer haustorium extract against alkoxyl radical-induced cytotoxicity by improving glutathione metabolism by modulating nrf2/haeme oxygenase-1 expression. J Am Coll Nutr.

[B22] Job JT, Rajagopal R, Alfarhan A, Narayanankutty A (2022b). Borassus flabellifer Linn haustorium methanol extract mitigates fluoride-induced apoptosis by enhancing Nrf2/Haeme oxygenase 1–dependent glutathione metabolism in intestinal epithelial cells. Drug Chem Toxicol.

[B23] Jomova K, Jenisova Z, Feszterova M, Baros S, Liska J, Hudecova D, Rhodes C, Valko M (2011). Arsenic: toxicity, oxidative stress and human disease. J Appl Toxicol.

[B24] Kang J, Chen J, Shi Y, Jia J, Zhang Y (2005). Curcumin-induced histone hypoacetylation: the role of reactive oxygen species. Biochem Pharmacol.

[B25] Laparra JM, Vélez D, Barberá R, Granero L, Polache A, Montoro R, Farré R (2006). Cytotoxic effect of As (III) in Caco-2 cells and evaluation of its human intestinal permeability. Toxicol in Vitro.

[B26] Lee H-I, McGregor RA, Choi M-S, Seo K-I, Jung UJ, Yeo J, Kim M-J, Lee M-K (2013). Low doses of curcumin protect alcohol-induced liver damage by modulation of the alcohol metabolic pathway, CYP2E1 and AMPK. Life Sci.

[B27] Liu J, Waalkes MP (2008). Liver is a target of arsenic carcinogenesis. Toxicol Sci.

[B28] Malayil D, House NC, Puthenparambil D, Job JT, Narayanankutty A (2022). Borassus flabellifer haustorium extract prevents pro-oxidant mediated cell death and LPS-induced inflammation. Drug Chem Toxicol.

[B29] Mirzaei H, Naseri G, Rezaee R, Mohammadi M, Banikazemi Z, Mirzaei HR, Salehi H, Peyvandi M, Pawelek JM, Sahebkar A (2016). Curcumin: A new candidate for melanoma therapy?. Int J Cancer.

[B30] Moghaddam NSA, Oskouie MN, Butler AE, Petit PX, Barreto GE, Sahebkar A (2019). Hormetic effects of curcumin: What is the evidence?. J Cell Physiol.

[B31] Moldeus P, Hogberg J, Orrenius S (1978). Isolation and use of liver cells. Methods Enzymol.

[B32] Morisco F, Vitaglione P, Amoruso D, Russo B, Fogliano V, Caporaso N (2008). Foods and liver health. Mol Asp Med.

[B33] Najafi N, Rezaee R, Hayes AW, Karimi G (2022). A review of mechanisms underlying the protective effects of natural compounds against arsenic-induced neurotoxicity. Biometals.

[B34] Narayanankutty A, Job JT, Narayanankutty V (2019). Glutathione, an antioxidant tripeptide: Dual roles in carcinogenesis and chemoprevention. Curr Protein Pept Sci.

[B35] Narayanankutty A, Nair A, Illam SP, Upaganlawar A, Raghavamenon AC (2021). Curcumin enriched VCO protects against 7,12-Dimethyl Benz[a] anthracene-induced skin papilloma in mice. Nutr Cancer.

[B36] Rezaee R, Momtazi AA, Monemi A, Sahebkar A (2017). Curcumin: a potentially powerful tool to reverse cisplatin-induced toxicity. Pharmacol Res.

[B37] Shahraki J, Motallebi A, Aghvami M, Pourahmad J (2013a). Ichthyotoxic cochlodinium polykrikoides induces mitochondrial mediated oxidative stress and apoptosis in rat liver hepatocytes. Iran J Pharm Res.

[B38] Shahraki J, Motallebi A, Pourahmad J (2013b). Oxidative mechanisms of fish hepatocyte toxicity by the harmful dinoflagellate Cochlodinium polykrikoides. Mar Environ Res.

[B39] Shahraki J, Zareh M, Kamalinejad M, Pourahmad J (2014). Cytoprotective effects of hydrophilic and lipophilic extracts of pistacia vera against oxidative versus carbonyl stress in rat hepatocytes. Iran J Pharm Res.

[B40] Sharma R, Gescher A, Steward W (2005). Curcumin: the story so far. Eur J Cancer.

[B41] Shi H, Shi X, Liu KJ (2004). Oxidative mechanism of arsenic toxicity and carcinogenesis. Mol Cell Biochem.

[B42] Stamatelos SK, Androulakis IP, Kong A-NT, Georgopoulos PG (2013). A semi-mechanistic integrated toxicokinetic–toxicodynamic (TK/TD) model for arsenic (III) in hepatocytes. J Theor Biol.

[B43] Susan A, Rajendran K, Sathyasivam K, Krishnan UM (2019). An overview of plant-based interventions to ameliorate arsenic toxicity. Biomed Pharmacother.

[B44] Tabrizian K, Musavi S, Rigi M, Hosseindadi F, Kordi S, Shamshirgaran F, Bazi A, Shahraki J, Rezaee R, Hashemzaei M (2019). Behavioral and molecular effects of intrahippocampal infusion of auraptene, resveratrol, and curcumin on H-89-induced deficits on spatial memory acquisition and retention in Morris water maze. Hum Exp Toxicol.

[B45] Wilken R, Veena MS, Wang MB, Srivatsan ES (2011). Curcumin: A review of anti-cancer properties and therapeutic activity in head and neck squamous cell carcinoma. Mol Cancer.

[B46] Xu Y, Wang Y, Zheng Q, Li X, Li B, Jin Y, Sun X, Sun G (2008). Association of oxidative stress with arsenic methylation in chronic arsenic-exposed children and adults. Toxicol Appl Pharmacol.

[B47] Yeang HXA, Hamdam JM, Al-Huseini LM, Sethu S, Djouhri L, Walsh J, Kitteringham N, Park BK, Goldring CE, Sathish JG (2012). Loss of transcription factor nuclear factor-erythroid 2 (NF-E2) p45-related factor-2 (Nrf2) leads to dysregulation of immune functions, redox homeostasis, and intracellular signaling in dendritic cells. J Biol Chem.

